# Neutrophil circadian rhythm is associated with different outcomes of acute kidney injury due to cholesterol crystal embolism

**DOI:** 10.3389/fcvm.2022.974759

**Published:** 2022-07-28

**Authors:** Chongxu Shi, Danyang Zhao, Lyuben Lyubenov, Manga Motrapu, Na Li, Stefanie Steiger, Elmina Mammadova-Bach, Luying Yang, Dong Liu, Hans-Joachim Anders

**Affiliations:** ^1^Nantong Laboratory of Development and Diseases, School of Life Sciences, Medical College, Nantong University, Nantong, China; ^2^Renal Division, Medizinische Klinik und Poliklinik IV, Klinikum der Universität München, Ludwig-Maximilians University Munich, Munich, Germany; ^3^Department of Nephrology, Center of Kidney and Urology, The Seventh Affiliated Hospital, Sun Yat-sen University, Shenzhen, China; ^4^Co-Innovation Center of Neuroregeneration, Key Laboratory of Neuroregeneration of Jiangsu and Ministry of Education, Nantong University, Nantong, China

**Keywords:** circadian rhythm, thrombosis, neutrophil, ischemic AKI, cholesterol crystal embolism (CCE)

## Abstract

Cholesterol crystal (CC) embolism can cause acute tissue infarction and ischemic necrosis via triggering diffuse thrombotic angiopathy occluding arterioles and arteries. Neutrophils contribute to crystal-induced immunothrombosis as well as to ischemic necrosis-related necroinflammation. We speculated that CC embolism-induced acute kidney injury (AKI) would be circadian rhythm-dependent and associated with cyclic differences in neutrophil function. Injection of CC into the left kidney induced thrombotic angiopathy progressing starting as early as 3 h after CC injection followed by a progressive ischemic cortical necrosis and AKI at 24 h. In C57BL/6J mice, circulating CD11b^+^Ly6G^+^ neutrophils were higher during the day phase [Zeitgeber time (ZT) 0–12] compared to the dark phase (ZT12-24). In the time frame of thrombus formation at ZT13, more neutrophils were recruited into the injured kidney 24 h later compared to CC embolism at ZT5. This effect was associated with an increased circulating number of CXCR2^+^ neutrophils as well as an upregulated kidney adhesion molecule and chemokine expression. These findings were associated with a significant increase in kidney necrosis, and endothelial injury at ZT13. Thus, the time of day has an effect also on CC embolism-related AKI in association with the circadian rhythm of neutrophil recruitment.

## Introduction

Atherosclerosis is a leading cause of global morbidity and mortality (1). Occlusive complications of atherosclerosis include atherothrombosis, atheroembolism, and cholesterol crystal (CC) embolism (1–3). CC embolism occurs at a frequency of 6.2 cases per million population per year and can be fatal (3, 4). Experimental CC embolism allows studying the pathophysiology of CC embolism-related ischemic cortical necrosis (5).

The circadian rhythm regulates behavior and physiological actions according to environmental changes, such as the adjustment of sleep-wake cycles, feeding, body temperature, blood pressure, heart rate, hormone secretion, metabolism (including lipid metabolism), and many other biological activities (6, 7). In the murine peripheral blood, numbers of leukocytes fluctuate with a peak at ZT5 (where ZT0 refers to lights on and ZT12 to lights off) (8). Leukocyte numbers show a peak at the beginning of the active phase (ZT13) in different organs, such as bone marrow, skeletal muscle, or the heart, which oscillate inversely with the blood (8). These fluctuations in immune cell trafficking into tissues coincide with sensitivity to acute inflammatory stimuli, being highest at the beginning of the active phase (9).

The effect of circadian rhythms on leukocyte functions has been studied in acute myocardial infarction, ischemic stroke, and arrhythmias also because the onset of the pathological events occur predominately during the sleep-to-wake transition period in humans (10–12). In humans, blood neutrophils oscillate throughout the day with a peak around 8:30 pm (13). These fluctuations in immune cell trafficking into tissues coincide with sensitivity to acute inflammatory stimuli, being highest at the beginning of the active phase (9).

Moreover, neutrophils infiltrate the heart also following a diurnal rhythm even under steady-state conditions (14). This circadian rhythm-dependent migration of neutrophils into the heart is regulated by CXCR2, the chemokine receptor rhythmic expressed on the neutrophils, and displayed a peak in the evening which is consistent with higher expression of *Icam-1, Vcam-1*, and the chemokine ligands in cardiac tissue (15). Consequently, during the active phase, an ischemic event leads to a strong inflammatory response and more injury, whereas lower neutrophil numbers reduce infarct size and maintain better cardiac function (16).

However, how oscillations in immune cell activity effect ischemic kidney injury or the consequences of CC embolism are unknown.

We report here the first experimental evidence that a circadian rhythms also affect CC embolism-related ischemic kidney injury and kidney function and propose an functional role of neutrophils in this pathological process.

## Materials and methods

### Preparation of CC injection solution

2 mg/ml of CC (grade ≥99%, Sigma Aldrich, Steinheim, Germany) solution was prepared with sterile phosphate-buffered saline (PBS) (5). The prepared solution was filtered through 25-33G needles multiple times to avoid clumping of the crystals. The concentration was calculated after filtration. Finally, the solution was autoclaved at 120°C and stored at 4°C. The injected concentration of CC was 10 mg/kg in all experiments, and the solution was warmed to body temperature and vortex shortly before use.

### Animal studies

CC embolism develops predominately in males (3), hence, we used only male mice in this study. However, we previously reported that male and female mice developed identical outcomes upon CC embolism (5). Seven to eight-week-old male healthy C57BL/6J mice were obtained from Charles River Laboratories (Sulzfeld, Germany) and housed in groups of *five* in polypropylene cages under a 12 h light/dark cycle, room temperature of 22 ± 2°C with unlimited access to food and water. The group size was 10 for different ZT experiments and calculated for glomerular filtration rate (GFR) as a primary endpoint and based on assumptions derived from previous experiments with this model. Animals were randomized by block randomization. Regular animal welfare scoring was performed according to regulations and procedures set by the local animal welfare committee. All experimental procedures were approved by the local government authorities according to the European equivalent of the NIH's Guide for the Care and Use of Laboratory Animals (directive 2010/63/EU). 66 mice were used for this study.

### Intraarterial injection of cholesterol crystals

CC injection was performed at different ZT. In brief: Mice were anesthetized by intraperitoneal injection of medetomidine (0.5 mg/kg), midazolam (5 mg/kg), and fentanyl (0.05 mg/kg) (17). After exposing the left kidney, 10 mg/kg of the warmed CC solution was injected via a 33G needle. Closing the wound with absorbable sutures after stopping the bleeding with a hemostatic gelatin sponge at the injection site. Administration of atipamezole (2.5 mg/kg) and flumazenil (0.5 mg/kg) antagonized anesthesia. Regular subcutaneous injections of buprenorphine were given as preemptive and post-interventional pain control.

### Measured glomerular filtration rate

We measured GFR transcutaneously in conscious and freely moving mice as described (17). Briefly: The mice were anesthetized by isofluorane inhalation for 10 s then a miniaturized imager device was attached to the mouse's back skin to record the signal, the device consisting of two light-emitting diodes, a photodiode, and a battery (MediBeacon, Mannheim, Germany). Firstly, record the background signal for ~5 min, then mice received I.V. injection of FITC-sinistrin (150 mg/kg) tracer (Mannheim Pharma & Diagnostics GmbH). Recording the FITC signal in mice blood for 1.5 h. The signal was analyzed using the MPD Lab Software (Mannheim Pharma & Diagnostics GmbH). The GFR was calculated by determining the decline of fluorescence intensity over time, representing the clearance of FITC-sinistrin via the kidney, and a model consisting of three compartments, mouse body weight, and an empirical conversion factor.

### Histological assessment

Twenty four hours post CC embolism, mice were sacrificed by cervical dislocation. Kidneys were harvested for immunostaining using standard protocols. Sections of 2 μm-thick kidney tissue were stained for fibrinogen (ab27913, Abcam) and smooth muscle actin (SMA, M0852, Dako) to localize arterial vessels and clots, respectively. Anti-CD31 (DIA-310, Dianova) was used to determine the approximate percentage of viable endothelial cells in the kidney. A semiquantitative injury score was assessed on periodic acid-Schiff (PAS)-stained sections. Injury criteria included the degree of tubular necrosis, tubular dilation, cast formation, brush border loss, and interstitial edema. Anti-Ly6B2+ (MCA771G, Serotec) was used to identify neutrophil infiltration into the renal parenchyma. All sections were assessed by a blinded observer.

### _Quantitative real-time PCR (qRT-PCR)_

One part of the kidneys (included cortex and outer medulla) was stored in RNA later solution at −20°C for RNA isolation. RNA was extracted from kidney tissue using Pure Link RNA Mini Kit (Invitrogen™, Germany) according to the manufacturer's protocol (5, 18). cDNA was synthesized from 2 μg of total RNA using the transcript kit (Invitrogen™, Germany). Quantitative real-time PCR (qRT-PCR) from cDNA was performed using the SYBR Green dye detection system on a Light Cycler 480 (Roche, Germany). Mouse samples were normalized to 18s rRNA. The sequences of gene-specific primers (300 nM; Metabion, Martinsried, Germany) are listed in Table 1.

**Table 1:** Mouse primers used in quantitative real-time PCR.

**Mouse gene**	**Forward primer 5**′**-3**′	**Reverse primer 3**′**-5**′
*Vcam-1*	GCTATGAGGATGGAAGACTCTGG	ACTTGTGCAGCCACCTGAGATC
*Icam-1*	AAACCAGACCCTGGAACTGCAC	GCCTGGCATTTCAGAGTCTGCT
*Cxcr2*	CCTCAAACGGGATGTATT	GCTCTGTCACCGATGTCT
*Cxcl1*	TCCAGAGCTTGAAGGTGTTGCC	AACCAAGGGAGCTTCAGGGTCA
*18s*	GCAATTATTCCCCATGAACG	AGGGCCTCACTAAACCATCC

*Icam-1, intercellular adhesion molecule 1; Vcam-1, vascular cell adhesion molecule 1; Cxcr2, CXC chemokine receptor 2; Cxcl1, CXC motif chemokine ligand 1*.

### Cytokine and chemokine analysis

Fifty microliter blood was collected by mouse tail vein, CXCL1 and CXCL12 in plasma supernatant were quantified with ELISA DuoSets from R&D systems.

### Neutrophil isolation from the peripheral blood

Twenty microlitre blood was collected by mouse tail vein, then added the same amount of cold 1.25% Dextran and kept at 4°C for 20–30 min to sediment. We carefully transferred the yellowish-orange supernatant into a 15 ml falcon and washed the leukocyte-rich supernatant with ice-cold 1x PBS. After discarding the supernatant, the pellet was resuspended with 1 ml of ice-cold 1x PBS. Followed by adding 12 ml of ice-cold sterile H2O to lysis the RBC (wait for the 20 s). Then 5 ml of ice-cold 0,6M KCl (keep at 4°C) was added to neutralize the solution (gently mix) and then washed with ice-cold 1x PBS twice. After adding the same volume of Ficoll-Hypaque into a 15 ml falcon, we carefully layered the cell suspension on the Ficoll-Hypaque. After centrifugation, the cell pellet was washed with ice-cold 1x PBS twice. Finally, resuspended the pellet with 1 ml of RPMI medium and counted the cells' concentration.

### Flow cytometry analysis of neutrophil numbers

The purified neutrophil suspensions were centrifuged, resuspended in FACS buffer (1x PBS with 1% BSA), and blocked with anti-mouse CD16/CD32 antibody (Fcγ III/II, BD Biosciences) for 10 min on ice. After blocking, cells were incubated with the following monoclonal antibodies for 30 min at 4°C in the dark: PE-Cy7 anti-mouse CD11b (clone M1/70, BioLegend), FITC anti-mouse Ly6G (clone 1A8, Biolegend), Percp-Cy5.5 anti-mouse CXCR2 (clone TG11, Biolegend), APC anti-mouse CXCR4 (clone 2B11, BD Bioscience). Data were acquired on a FACS Canto II (BD Biosciences), and analysis was performed with FlowJo software (Ashland, USA). Neutrophils were identified as CD11b^+^Ly6G^+^, CXCR2^+^/CXCR4^−^ cells were fresh neutrophils, and CXCR2^−^/CXCR4^+^ cells were aged neutrophils.

### Statistical analysis

All statistical analyses were performed using GraphPad Prism 8.0 Software (GraphPad Software, San Diego, USA). Prior to each analysis, we assessed the normal distribution of the data (Shapiro-Wilk test), homo- and heteroscedasticity (Levene's test), and the presence of outliers (Grubb's test). Normally distributed and homoscedastic data were tested for statistical significance using a one-way ANOVA (3 or more groups) and *t*-test (2 groups). The *post-doc* Bonferroni test was used for multiple comparisons. Heteroscedastic data were corrected using the Games-Howell *post-hoc* test. Not normally distributed data sets were compared using the Kruskal-Wallis test. A *p* < 0.05 was considered statistically significant.

## Results

### Development of arterial crystal clots and necroinflammation after CC embolism

**Figure 1:**
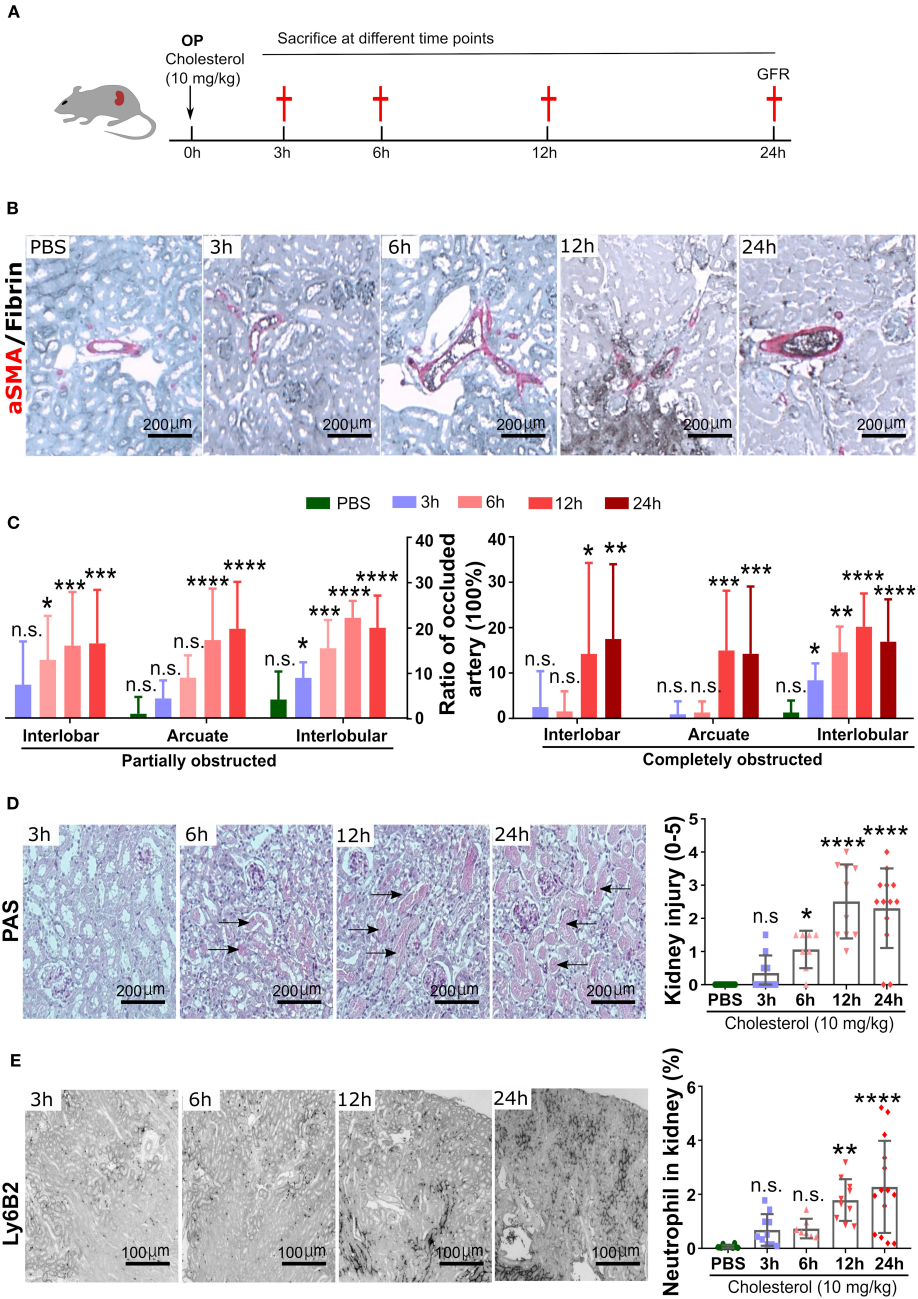
Time kinetics of crystal clots formation and growth. **(A)** Schematic of experiment. **(B)** Representative images of aSMA/Fibrin staining at 3, 6, 12, and 24 h post CC injection and PBS control. **(C)** Partially and completely obstructed arteries in the kidney 24 h post-CC injection (*n* = 8–12 mice/group). **(D)** Representative images of PAS staining at 3, 6, 12, and 24 h post-CC injection, and kidney injury score (*n* = 8–12 mice/group). **(E)** Representative images of Ly6B2+ staining at 3, 6, 12, and 24 h post-CC injection, and neutrophils percentage in the kidney (*n* = 8–14 mice/group). Data are means ± *SD*. One-way ANOVA. *P < 0.05; **P < 0.01; ***P < 0.001. ****P < 0.001.

Experimental kidney CC embolism caused a diffuse thrombotic arteriopathy, AKI, and ischemic cortical necrosis with a perilesional inflammatory reaction known from territorial infarcts in other organs such as myocardial infarction or ischemic stroke (3, 5, 19). We first characterized the dynamics of crystal clot formation triggered by CC inside renal arteries, C57BL/6J WT mice were sacrificed at different time points upon CC embolism (Figure 1A). Immunostaining for aSMA/Fibrin showed arterial thrombus formation starting as early as 3 h after CC-injection (Figure 1B). As CC embolism progressed with time, the percentage of complete/partial occlusions increased from 3 to 12 h after CC injection (Figure 1C). The finding of progressively increasing complete arterial occlusions mechanistically explained the progressive ischemic kidney necrosis (Figure 1D), which also reached a plateau at 12 h after CC embolism. In contrast, infiltration of Ly6B.2+ immune cells, which represented the dominant immune cell population (neutrophils) at this time point, continued to increase up to 24 h after CC embolism (Figure 1E). Thus, CC embolism induces obstructive thrombotic angiopathy and ischemic tissue necrosis within 3–12 h, associated with lesion and perilesional immune cell recruitment that continues to progress beyond 12 h, i.e., tissue necroinflammation.

### Circadian oscillations of circulating blood neutrophils under steady-state

**Figure 2:**
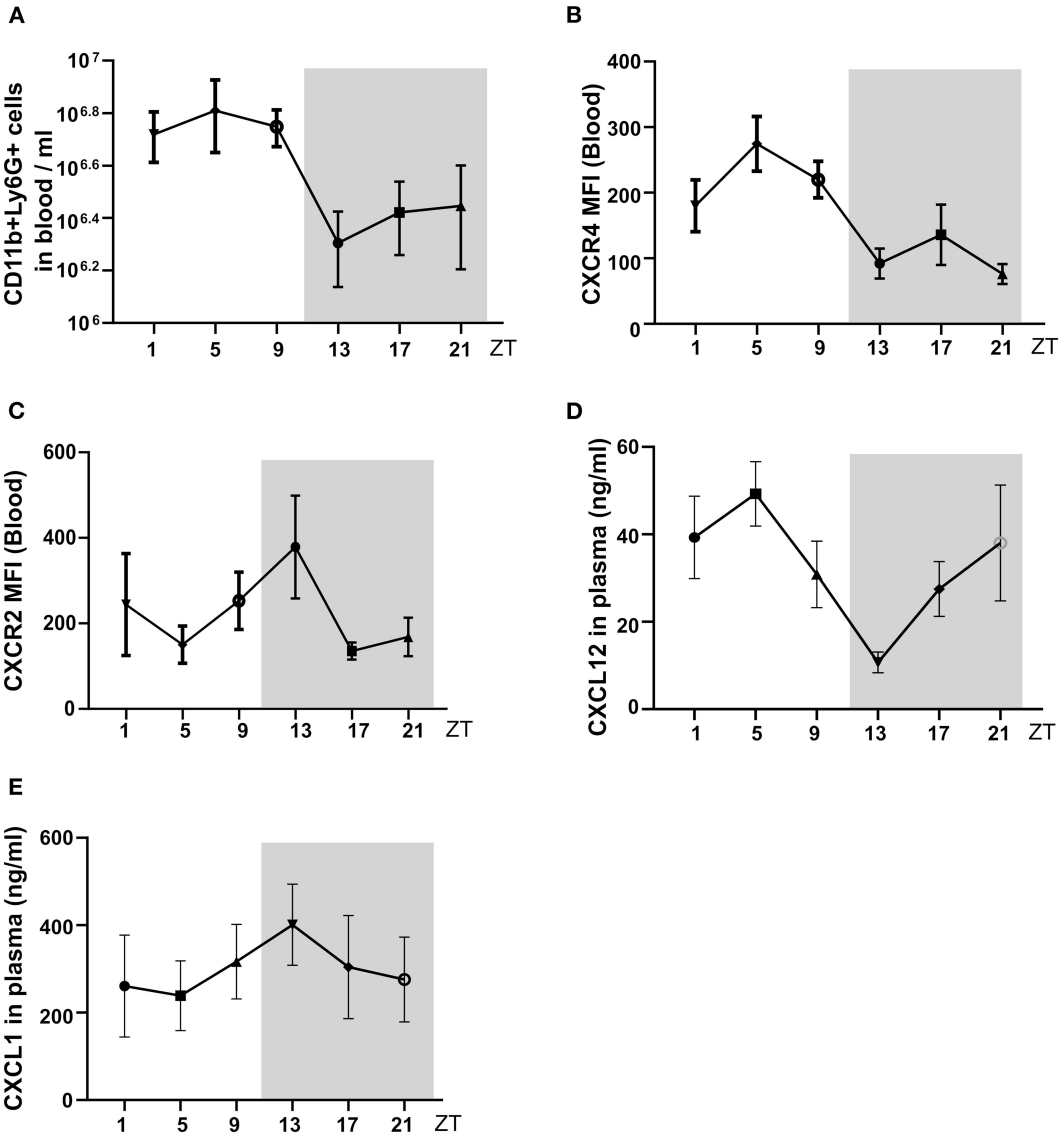
The circadian oscillations of neutrophils, and chemokine in mouse blood in a healthy state. **(A)** Flow cytometric quantification of neutrophils baseline in the blood. **(B,C)** MFI of surface CXCR4 (**B**, *n* = 5 mice/group) and CXCR2 (**C**, *n* = 5 mice/group) expression by neutrophils in the blood as determined by flow cytometry. **(D,E)** Plasma levels of CXCL12 (**D**, *n* = 5 mice/group) and CXCL1 (**E**, *n* = 5 mice/group) in healthy mice as determined by ELISA. Data are means ± *SD*. MFI: mean fluorescence intensity; CXCR2, CXC chemokine receptor 2; CXCR4, CXC chemokine receptor 4; CXCL12, CXC motif chemokine ligand 12.

Next, we tested for circadian changes in neutrophil counts in the peripheral blood in C57BL/6J mice, as previously reported (8, 20). The analysis of blood counts in healthy control C57BL/6J mice revealed a peak of blood total neutrophil counts at ZT5 and the lowest levels around ZT13 (Figure 2A). This was consistent with the numbers of CXCR4^+^ “aged” neutrophils that also peaked at ZT5 and were lowest at ZT13 (Figure 2B). In contrast, the CXCR2^+^ “young” neutrophils peaked at ZT13 and were lowest at ZT5 (Figure 2C). Circulating levels of the neutrophil homing signal CXCL1 increased from ZT5 to ZT13 (Figure 2E), and CXCL12 levels were decreased from ZT5 to ZT13 (Figure 2D). Therefore, in C57BL/6J mice, neutrophil numbers, and their chemokines follow a circadian rhythm with a peak during the day. Notably, neural signals appear to control the regulation of CXCL12 levels in the mouse bone marrow (BM). Sympathetic nerves inhibit CXCL12 expression and generate oscillatory expression of the chemokine, because it innervates the BM deliver diurnal adrenergic signals to stromal cells through β3-adrenergic receptors (21). While cholinergic signals from the parasympathetic nervous system inhibit adrenergic activity of the murine sympathetic nervous system at night (22), altogether establishing tight temporal patterns in the BM.

### Circadian oscillations of mouse GFR and kidney *Icam, Vcam, Cxcl1*, and *Cxcr2* mRNA expression in healthy mice

**Figure 3:**
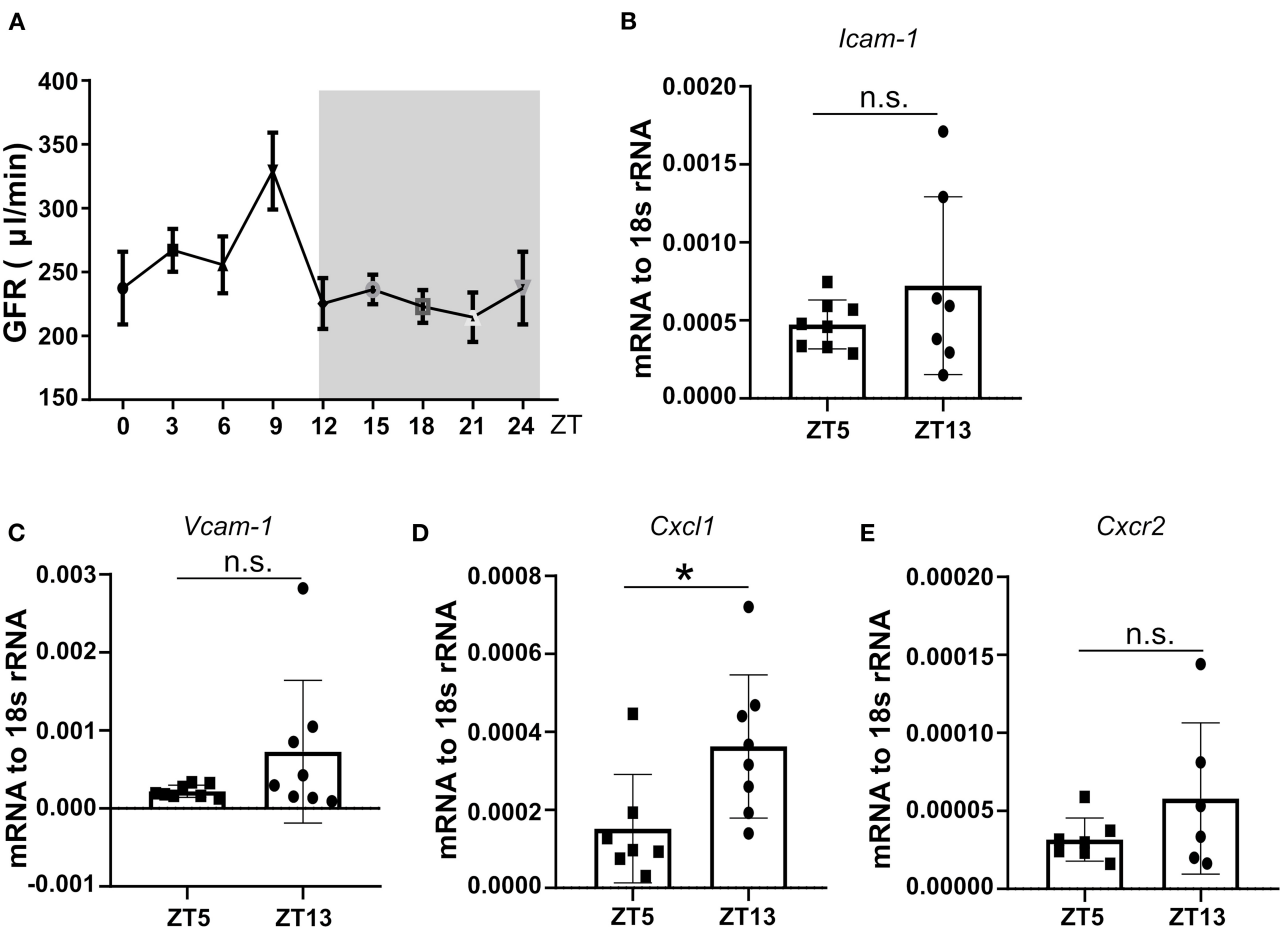
The circadian oscillation of mouse GFR, kidney mRNA expression levels of adhesion molecules, and chemokines at ZT5 and ZT13 in a healthy state. **(A)** Circadian oscillations of mouse GFR in the healthy state (*n* = 8–10 mice/group). **(B,C)** mRNA expression of adhesion molecule *Icam-1* (**B**, *n* = 7–8 mice/group) and *Vcam-1* (**C**, *n* = 7–8 mice/group) in mice kidney at ZT5 and ZT13. **(D)** mRNA expression of neutrophil mediating chemotaxis *Cxcl1* (**D**, *n* = 7–8 mice/group) and its receptor *Cxcr2* (**E**, *n* = 6–7 mice/group) at ZT5 and ZT13. *Icam-1*, intercellular adhesion molecule 1; *Vcam-1*, vascular cell adhesion molecule 1; *Cxcr2*, CXC chemokine receptor 2; *Cxcl1*, CXC motif chemokine ligand 1. While background: daytime, gray background: nighttime. Data are means ± *SD*, unpaired Student's *t*-test, *P < 0.05.

We followed neutrophil numbers, and their chemokine levels in mouse circadian rhythm and detected peaks during the day. First, mouse GFR at different ZT times under a steady-state and found the GFR showed rhythmic change during the day (Figure 3A). Furthermore, we found that mRNA expression of kidney immune cell adhesion molecule *Icam-1* and *Vcam-1* at ZT13 was higher than ZT5 (Figures 3B,C), which was paralleled by enhanced mRNA levels of *Cxcl1* chemokines thereby modulating neutrophil chemotaxis (Figure 3D). Consequently, the analysis of the corresponding chemokine receptor *Cxcr2* for *Cxcl1* in the kidney revealed a higher expression level at ZT13 in the kidney (Figure 3E). These results suggest that in healthy mice, neutrophil trafficking and effector functions are regulated by the circadian rhythm in the kidney and this process may have a pathological impact on CC embolism.

### The active phase of circadian rhythm exacerbates kidney injury during CC embolism

**Figure 4:**
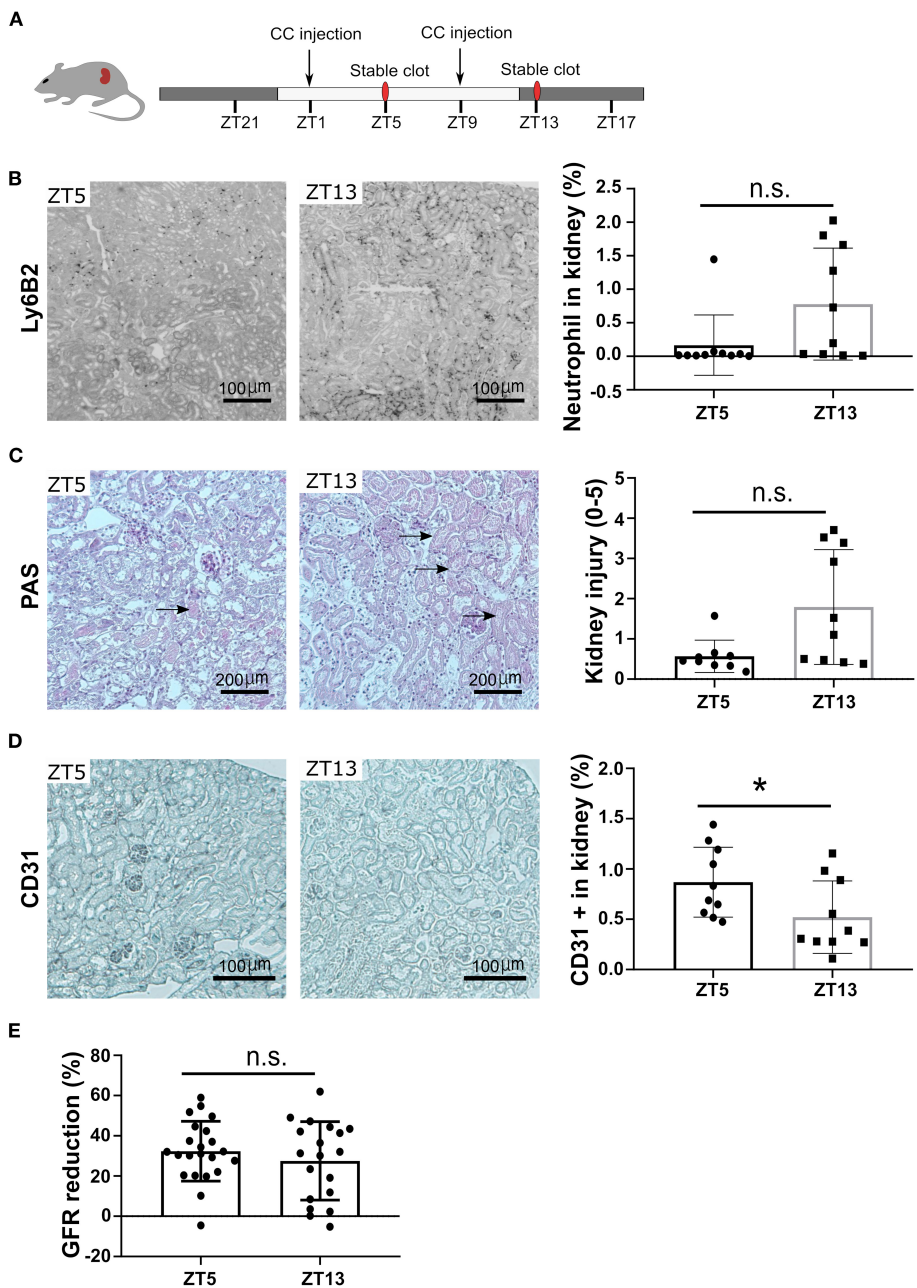
CC embolism during the active phase leads to more kidney injury. **(A)** Schematic of experiment. **(B)** Representative images of Ly6B2+ staining, and neutrophil counts in the kidney at ZT5 and ZT13 (*n* = 10 mice/group). **(C)** Representative images of PAS staining, and mouse kidney injury score at ZT5 and ZT13 (*n* = 10 mice/group). **(D)** Representative images of CD31 staining, and endothelial injury in the kidney (*n* = 10 mice/group). **(E)** GFR reduction upon ZT5 and ZT13 24 h post-CC injection (*n* = 20 mice/group). While background: daytime, gray background: nighttime. Data are means ± *SD*, unpaired Student's *t*-test, *P < 0.05.

To follow the pathophysiological function of the circadian rhythm-induced CXCR2^+^ neutrophil recruitment in the kidney, we performed CC embolism at different time points. According to the above-indicated results from time kinetics of crystal clot formation, we injected CC at ZT1 and ZT9 to study the pathological impact of different neutrophil numbers on CC-induced ischemic kidney necrosis at ZT5 and ZT13, respectively (Figure 4A). Interestingly, mice subjected to CC embolism at ZT13 had higher neutrophil counts in the ischemic area 24 h post-CC embolism compared to ZT5 (Figure 4B). In line with the enhanced neutrophil infiltration after ZT13 CC embolism, kidney tubular injury tended to be higher in mice with ZT13 compared to ZT5 (24 h post-CCE), albeit the differences were not significant (Figure 4C). Consistent with the increased levels of neutrophils, the CC-injected kidney showed significantly increased endothelial injury at ZT13 (Figure 4D). Of note, the GFR reduction 24 h post-CC injection did not differ between ZT5 and ZT13 (Figure 4E). These results suggest that time of day had an effect on CC embolism-induced ischemic kidney necrosis, endothelial injury, and neutrophil infiltration but not in GFR loss.

### Enhanced neutrophil recruitment relates to high CXCR2 expression in the kidney

**Figure 5:**
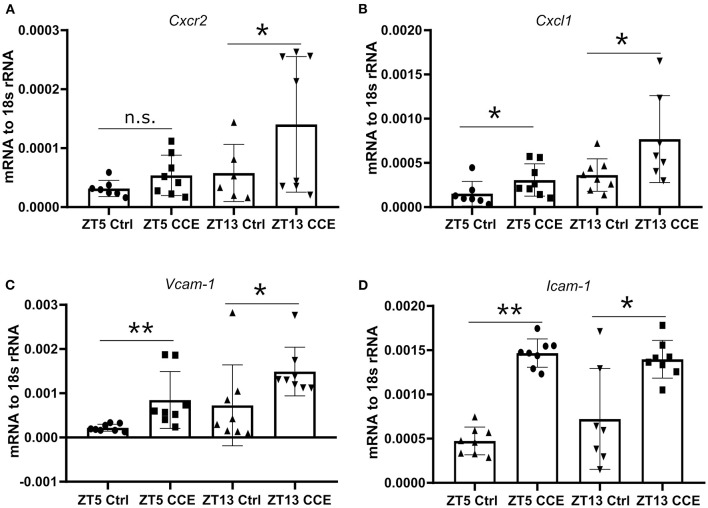
Enhanced kidney neutrophil recruitment is CXCR2 dependent. The intrarenal mRNA expression level of *Cxcr2* (**A**, *n* = 6–8 mice/group), *Cxcl1* (**B**, *n* = 7–8 mice/group), *Vcam-1* (**C**, *n* = 7–8 mice/group), and *Icam-1* (**D**, *n* = 7–8 mice/group) at ZT5 and ZT13 before and post-CC injection. Data are means ± *SD*. *Icam-1*, intercellular adhesion molecule 1; *Vcam-1*, vascular cell adhesion molecule 1; *Cxcr2*, CXC chemokine receptor 2; *Cxcl1*, CXC motif chemokine ligand 1. Unpaired Student's *t*-test, *P < 0.05, **P < 0.01.

To explore the underlying mechanisms of neutrophil recruitment to the kidney, we focused on the mRNA levels of *Cxcl1* and its corresponding chemokine receptor *Cxcr2*, as their expression levels in the kidney under a steady-state coincided with the peak of CXCR2^+^ neutrophils and CXCL1 chemokine in the circulation (Figures 2C,E). The circulating immune cell population was heterogeneous, composed of young and aged neutrophils, and the cell number is regulated by aging and replenishment from the bone marrow (23). The fresh neutrophils exhibit enhanced chemotactic activity and the ability to respond to inflammatory stimuli (24). Significantly elevated mRNA levels of *Cxcr2* (Figure 5A) and its ligands *Cxcl1* (Figure 5B) were found in CC-injected kidneys at ZT13 compared to ZT5. In addition, mRNA levels of *Icam-1* (Figure 5C), and *Vcam-1* (Figure 5D) in mice kidneys were also highly increased 24 h post CC injection. These data suggest that circadian rhythm-dependent differences in the migration of neutrophils into the kidney may be regulated by differences in levels of CXCR2 expression.

## Discussion

**Figure 6:**
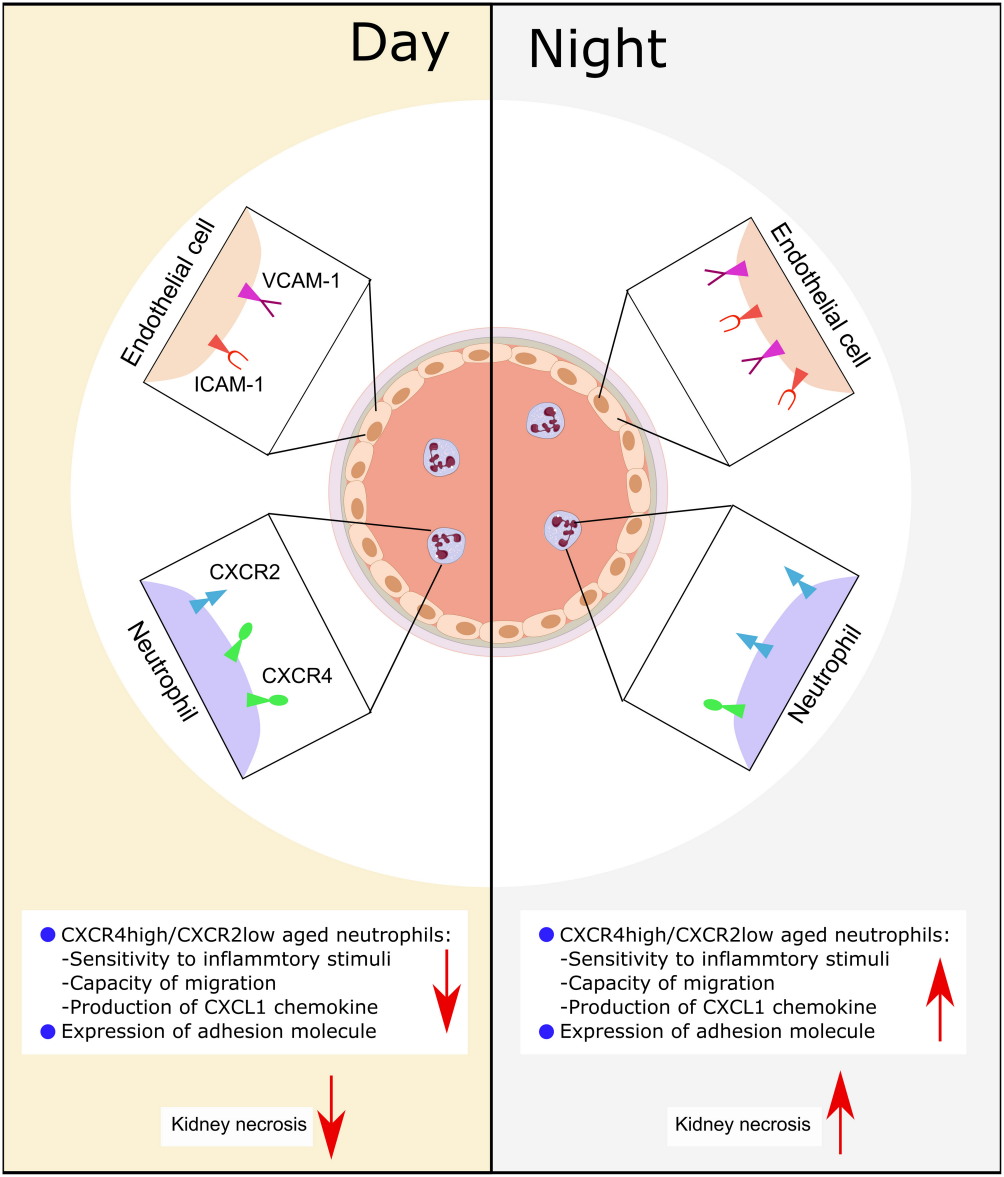
Schematic illustration of the putative mechanism of circadian rhythm-related kidney injury. During the day, the expression of adhesion molecule (*Icam*-1, *Vcam*-1) on endothelial is lower than the night, the release of chemokine (CXCL1) from immune cells is less than the night, the expression of CXCR2 is lower, while the CXCR4 is high. Consequently, this leads to more tissue necrosis during the night.

We had hypothesized that circadian rhythm could potentially modulate neutrophil recruitment to the kidney after CC embolism. Using a novel CC embolism model in mice, we found that: (a) CC embolism-induced arterial obstructions gradually increased within 3–12 h after CC embolism in the kidney, and followed by progressive ischemic tubular injury up to 24 h, (b) Time of day had an effect on healthy WT mice GFR, circulating neutrophils and their recruitment to the injured kidney, (c) Differences in CXCR2 receptor expression on neutrophils and its respective ligand in the injured kidney may explain these differences. These findings imply that time of day may determine the outcomes of CC embolism (Figure 6).

In this project, we investigated the time kinetics of crystal clot formation and found that crystal clots as early as 3 h after CC embolism. We assume that CC impacting the endothelial lining of kidney arteries induces endothelial cell injury, and release of tissue factor and chromatin, both activating platelets (5). Activated platelets release pro-coagulant and pro-inflammatory mediators from their granules, thereby inducing thrombus growth as a gradual process at the site of injury (5, 25, 26). As with arterial thrombosis, in this pathological process, thrombus formation occurs at injured kidney vessel sites, which subsequently leads to the apposition of a mesh composed of fibrin and extracellular DNA to trap circulating platelets, neutrophils, and red blood cells (27, 28).

Previous studies suggest that young CXCR2^+^ neutrophils have enhanced pro-inflammatory properties and an increased migratory capacity (23). We found a high expression of CXCR2^+^ at ZT13 in steady-state when high numbers of aged CXCR4^+^ aged neutrophils were present in the circulation (23). In line with other reports, we found oscillations of neutrophil numbers in mice blood peaking at ZT5 and lowest at ZT13 under a steady-state (9). In support of these data, we also found enhanced kidney expression of adhesion molecules (*Icam-1, Vcam-1*) and fresh neutrophil chemoattractants *Cxcl1* and chemokine receptor *Cxcr2* at ZT13 in the kidney. Neutrophils represent the predominant innate immune cell population that massively infiltrates the injured tubular within the first hours. Consequently, if an ischemic injury occurs during the active phase, more CXCR2^+^ fresh neutrophils migrate to the kidney to respond locally to the ischemic injury by releasing pro-inflammatory mediators to attract more inflammatory cells into the area of the ischemic kidney necrosis. These process may lead to an uncontrollable inflammatory response and more severe kidney injury. Neutrophil-depletion studies might shed further light into this issue.

Neutrophil are produced within the bone marrow via the process of granulopoiesis. During steady-state, neutrophils patrol through many tissues in mice (29). Notably, infiltration of neutrophils into most naïve tissues follows circadian patterns with a peak at night (29). The pathophysiological consequence of the circadian oscillation of neutrophils in blood has been correlated with vascular disease and used for prognosis in the clinics (30). Aged neutrophils are preferentially cleared from the circulation into healthy tissues under steady-state conditions (15), whereas fresh neutrophils preferentially recruit to inflammatory sites (15). The rhythmic recruitment of fresh and aged neutrophils could be also responsible for the circadian manifestation of various inflammatory diseases. In a model of myocardial ischemia, the infiltration of neutrophils increased during the night (ZT13) aggravating cardiac injury (14). In this case, the differential recruitment was CXCR2-dependent (14). This correlates with data showing increased CXCR2 expression on neutrophils at night also in our model (15). Whether timed infiltration of different type neutrophils in this tissue influences other physiological organ functions remains to be explored.

Kidney CC embolism impairs kidney function in various progressive ways (31, 32). Acute onset occurs within 1 week of a clear causal trigger of atheroembolism and presents as loss of excretory kidney function in 20–30% of patients (31). This incident of AKI indicates diffuse intrarenal CCE. Subacute kidney disease, as the most frequent form of CC embolism, with accelerating kidney function loss arising progressively during several weeks of the triggering event. The chronic onset of organ failure suggests clinically silent CC embolism, usually remaining undiagnosed because kidney biopsy is not routinely performed. The rate of CKD progression is highly variable. 28–61% of patients at acute or subacute stages need kidney replacement therapy, and partial recovery of kidney function occurs in 20–30% of those patients after a variable period of dialysis (32). Recovery of kidney function is associated with reduced inflammatory response and resolution of concurrent acute tubular necrosis in ischemic areas. Moreover, kidney CC embolism also associates with severe, uncontrolled hypertension. Kidney infarction is a unique outcome of CC embolism (33).

Limitations of our study include the short period of the analysis and the specific focus on neutrophils. Similar circadian rhythm may apply to platelet functions (e.g., oszillations in serotonin uptake and release) or other cell types involved. As such, any interpretation of the role of neutrophils in the observed kidney phenotype is merely associative but not necessarily causative in this context. We could study only unilateral kidney embolism, although in clinical practice CC embolism usually affects both kidneys. However, technical and animal welfare aspects did not allow bilateral CC embolism.

In summary, our findings suggest that the time of day may determine CC-injection-related AKI and outcomes in association with circadian oscillations in neutrophil numbers, surface expression of chemokine receptors, and neutrophil recruitment to the injured kidney.

## Data availability statement

The original contributions presented in the study are included in the article/supplementary material, further inquiries can be directed to the corresponding author.

## Ethics statement

The animal study was reviewed and approved by Regierung von Oberbayern based on the European directive for the Protection of Animals Used for Scientific Purposes (2010/63/EU).

## Author contributions

CS and H-JA designed the study and experiments. CS, LL, and MM conducted experiments and analyzed data. CS and DZ performed microscopy of mouse tissue sections and analyzed data. NL and SS performed flow cytometry. EM-B and LY supported the analyses. CS, H-JA, and DL wrote the first draft of the manuscript. All authors edited the first manuscript draft and approved the submitted version.

## Conflict of interest

The authors declare that the research was conducted in the absence of any commercial or financial relationships that could be construed as a potential conflict of interest.

## Publisher's note

All claims expressed in this article are solely those of the authors and do not necessarily represent those of their affiliated organizations, or those of the publisher, the editors and the reviewers. Any product that may be evaluated in this article, or claim that may be made by its manufacturer, is not guaranteed or endorsed by the publisher.

## References

[1] Weber C Noels H. Atherosclerosis: current pathogenesis and therapeutic options. Nat Med. (2011) 17:1410–22. 10.1038/nm.2538 22064431

[2] Frangogiannis NG. Pathophysiology of myocardial infarction. Compr Physiol. (2015) 5:1841–75. 10.1002/cphy.c150006 26426469

[3] Scolari F Ravani P Divisione S Dialisi N. Atheroembolic renal disease. Lancet. (2010) 375:1650–60. 10.1016/S0140-6736(09)62073-0 20381857

[4] Moolenaar W. Cholesterol crystal embolization in the Netherlands. Arch Intern Med. (2011) 156:653–7. 10.1001/archinte.1996.00440060081009 8629877

[5] Shi C Kim T Steiger S Mulay SR Klinkhammer BM Bauerle T. Crystal clots as therapeutic target in cholesterol crystal embolism. Circ Res. (2020) 126:e37–52. 10.1161/CIRCRESAHA.119.315625 32089086

[6] Chen L Yang G. Recent advances in circadian rhythms in cardiovascular system. Front Pharmacol. (2015) 6:71. 10.3389/fphar.2015.00071 25883568 PMC4381645

[7] Feng D Lazar MA. Clocks, metabolism, and the epigenome. Mol Cell. (2012) 47:158–67. 10.1016/j.molcel.2012.06.026 22841001 PMC3408602

[8] Scheiermann C Kunisaki Y Lucas D Chow A Jang J-E Zhang D. Adrenergic nerves govern circadian leukocyte recruitment to tissues. Immunity. (2012) 37:290–301. 10.1016/j.immuni.2012.05.021 22863835 PMC3428436

[9] Scheiermann C Kunisaki Y Frenette PS. Circadian control of the immune system. Nat Rev Immunol. (2013) 13:190–8. 10.1038/nri3386 23391992 PMC4090048

[10] Muller JE Stone PH Turi ZG Rutherford JD Czeisler CA Parker C. Circadian variation in the frequency of onset of acute myocardial infarction. N Engl J Med. (1985) 313:1315–22. 10.1056/NEJM198511213132103 2865677

[11] Muller JE Ludmer PL Willich SN Tofler GH Aylmer G Klangos I. Circadian variation in the frequency of sudden cardiac death. Circulation. (1987) 75:131–8. 10.1161/01.CIR.75.1.131 3791599

[12] Muller JE Tofler GH Willich SN Stone PH. Circadian variation of cardiovascular disease and sympathetic activity. J Cardiovasc Pharmacol. (1987) 10:S104–9; discussion S110–1. 10.1097/00005344-198710011-00020 2481159

[13] Sennels HP Jørgensen HL Hansen A-LS Goetze JP Fahrenkrug J. Diurnal variation of hematology parameters in healthy young males: the Bispebjerg study of diurnal variations. Scand J Clin Lab Invest. (2011) 71:532–41. 10.3109/00365513.2011.602422 21988588

[14] Schloss MJ Horckmans M Nitz K Duchene J Drechsler M Bidzhekov K. The time-of-day of myocardial infarction onset affects healing through oscillations in cardiac neutrophil recruitment. EMBO Mol Med. (2016) 8:937–48. 10.15252/emmm.201506083 27226028 PMC4967945

[15] Adrover JM Del Fresno C Crainiciuc G Cuartero MI Casanova-Acebes M Weiss LA. A neutrophil timer coordinates immune defense and vascular protection. Immunity. (2019) 50:390–402.e10. 10.1016/j.immuni.2019.11.001 30709741

[16] Yamazaki S Numano R Abe M Hida A Takahashi R Ueda M. Resetting central and peripheral circadian oscillators in transgenic rats. Science. (2000) 288:682–5. 10.1126/science.288.5466.682 10784453

[17] Marschner JA Schäfer H Holderied A Anders H-J. Optimizing mouse surgery with online rectal temperature monitoring and preoperative heat supply. Effects on post-ischemic acute kidney injury. PLoS ONE. (2016) 11:e0149489. 10.1371/journal.pone.0149489 26890071 PMC4758659

[18] Gudsoorkar PS Thakar CV. Acute kidney injury, heart failure, and health outcomes. Cardiol Clin. (2019) 37:297–305. 10.1016/j.ccl.2019.04.005 31279423

[19] Laridan E Denorme F Desender L Francois O Andersson T Deckmyn H. Neutrophil extracellular traps in ischemic stroke thrombi. Ann Neurol. (2017) 82:223–32. 10.1002/ana.24993 28696508

[20] Schloss MJ Horckmans M Nitz K Duchene J Drechsler M Bidzhekov K. The time-of-day of myocardial infarction onset affects healing through oscillations in cardiac neutrophil recruitment. EMBO Mol Med. (2016) 8:937–48. 27226028 10.15252/emmm.201506083 PMC4967945

[21] Méndez-Ferrer S Lucas D Battista M Frenette PS. Haematopoietic stem cell release is regulated by circadian oscillations. Nature. (2008) 452:442–7. 10.1038/nature06685 18256599

[22] García-García A Korn C García-Fernández M Domingues O Villadiego J Martín-Pérez D. Dual cholinergic signals regulate daily migration of hematopoietic stem cells and leukocytes. Blood. (2019) 133:224–36. 10.1182/blood-2018-08-867648 30361261 PMC6449569

[23] Casanova-Acebes M Pitaval C Weiss LA Nombela-Arrieta C Chèvre R A-González N. Rhythmic modulation of the hematopoietic niche through neutrophil clearance. Cell. (2013) 153:1025–35. 10.1016/j.cell.2013.04.040 23706740 PMC4128329

[24] Whyte MK Meagher LC MacDermot J Haslett C. Impairment of function in aging neutrophils is associated with apoptosis. J Immunol. (1993) 150:5124–34. 8388425

[25] Falasca GF Ramachandrula A Kelley KA O'Connor CR Reginato AJ. Superoxide anion production and phagocytosis of crystals by cultured endothelial cells. Arthritis Rheum. (1993) 36:105–16. 10.1002/art.1780360118 8381009

[26] Nymo S Niyonzima N Espevik T Mollnes TE. Cholesterol crystal-induced endothelial cell activation is complement-dependent and mediated by TNF. Immunobiology. (2014) 219:786–92. 10.1016/j.imbio.2014.06.006 25053140

[27] Mackman N. Triggers, targets and treatments for thrombosis. Nature. (2008) 451:914–8. 10.1038/nature06797 18288180 PMC2848509

[28] Engelmann B Massberg S. Thrombosis as an intravascular effector of innate immunity. Nat Rev Immunol. (2013) 13:34–45. 10.1038/nri3345 23222502

[29] Casanova-Acebes M Nicolás-Ávila JA Li JL García-Silva S Balachander A Rubio-Ponce A. Neutrophils instruct homeostatic and pathological states in naive tissues. J Exp Med. (2018) 215:2778–95. 10.1084/jem.20181468 30282719 PMC6219739

[30] Coller BS. Leukocytosis and ischemic vascular disease morbidity and mortality: is it time to intervene? Arterioscler Thromb Vasc Biol. (2005) 25:658–70. 10.1161/01.ATV.0000156877.94472.a5 15662026

[31] Meyrier A. Cholesterol crystal embolism: diagnosis and treatment. Kidney Int. (2006) 69:1308–12. 10.1038/sj.ki.5000263 16614719

[32] Belenfant X Meyrier A Jacquot C. Supportive treatment improves survival in multivisceral cholesterol crystal embolism. Am J Kidney Dis. (1999) 33:840–50. 10.1016/S0272-6386(99)70415-4 10213638

[33] Lie JT. Cholesterol atheromatous embolism. The great masquerader revisited. Pathol Annu. (1992) 27:17–50. 1584626

